# Oxygen treatment for cluster headache attacks at different flow rates: a double-blind, randomized, crossover study

**DOI:** 10.1186/s10194-018-0917-4

**Published:** 2018-10-10

**Authors:** Thijs H. T. Dirkx, Danielle Y. P. Haane, Peter J. Koehler

**Affiliations:** Department of Neurology, Zuyderland Medical Center Heerlen, PO Box 4446, 6401 CX Heerlen, The Netherlands

**Keywords:** Cluster headache, Oxygen, Acute treatment, Randomized controlled trial

## Abstract

**Background:**

Cluster headache attacks can, in many patients, be successfully treated with oxygen via a non-rebreather mask. In previous studies oxygen at flow rates of both 7 L/min and 12 L/min was shown to be effective. The aim of this study was to compare the effect of 100% oxygen at different flow rates for the treatment of cluster headache attacks.

**Methods:**

In a double-blind, randomized, crossover study, oxygen naïve cluster headache patients, treated attacks with oxygen at 7 and 12 L/min. The primary outcome measure was the percentage of attacks after which patients (treating at least 2 attacks/day) were painfree after 15 min, in the first two days of the study. Secondary outcome measures were percentage of successfully treated attacks, percentage of attacks after which patients were painfree, drop in VAS score and patient preference in all treatment periods (14 days).

**Results:**

Ninety-eight patients were enrolled, 70 provided valid data, 56 used both flow rates. These 56 patients recorded 604 attacks, eligible for the primary analysis. An exploratory analysis was conducted using all eligible attacks of 70 patients who provided valid data. We could only include 5 patients, treating 27 attacks on the first two days of the study, for our primary outcome, which did not show a significant difference (*p* = 0.180). Patients tended to prefer 12 L/min (*p* = 0.005). Contradicting this result, more patients were painfree using 7 L/min (*p* = 0.039). There were no differences in side effects or in our other secondary outcome measures. The exploratory analysis showed an odds ratio of being painfree using 12 L/min of 0.73 (95% CI 0.52–1.02) compared to 7 L/min (*p* = 0.061) as scored on a 5-point scale. The average drop in score on this 5-point scale, however, was equal between groups. Also slightly more patients noticed, no or not much, relief on 7 L/min, and found 12 L/min to be effective in all their attacks.

**Conclusion:**

There is lack of evidence to support differences in the effect of oxygen at a flow rate of 12 L/min compared to 7 L/min. More patients were painfree using 7 L/min, but our other outcome measures did not confirm a difference in effect between flow rates. As most patients prefer 12 L/min and treatments were equally safe, this could be used in all patients. It might be more cost-effective, however, to start with 7 L/min and, if ineffective, to switch to 12 L/min.

**Trial registration:**

European Union Clinical Trials Register (2012–003648-59), registered 1 October 2012. Dutch Trial Register (NTR3801), registered 14 January 2013.

## Background

Cluster headache (CH) attacks can, in many patients, be successfully treated with oxygen via a non-rebreather mask [1–4]. A study by Kudrow (1981) (*N* = 52) demonstrated that 75% of patients treated with oxygen at a flow rate of 7 L/min have adequate or complete relief, in at least 7 out of 10 attacks [1]. In a second study by Kudrow (*N* = 50), oxygen at 7 L/min was effective in 82%, compared to 70% with ergotamine [1]. In a small study by Fogan (1985) (*N* = 19) oxygen at a flow rate of 6 L/min was shown to be more effective than room air [2]. The endpoint was a mean relief score (0 = no relief to 3 = complete relief). The average relief score for all oxygen-treated patients was 1.93, compared to 0.77 for room air.

The usual oxygen flow rate applied has remained 7 L/min until the study by Cohen (2009) (*N* = 76) showed that treatment with oxygen at a flow rate of 12 L/min was effective as well [3]. The CH attacks stopped, or adequate relief was obtained, within 15 min of oxygen usage in 78%, compared to 20% using room air. In this study the primary endpoint was to render the patient painfree or have adequate relief while treating a single attack. As this endpoint is very different from the one used by Kudrow and Fogan [1, 2], these studies are difficult to compare. A beneficial effect of oxygen at 14–15 L/min in patients not responding to 7–10 L/min has been described in a small case series of 3 patients [5].

A controlled study to compare different oxygen flow rates has not been conducted. The use of oxygen at a flow rate of 12 L/min without proper evidence of its superiority to 7 L/min seems inefficient, considering the additional costs of production and delivery. In the present study we compared treatment of CH attacks with 100% oxygen via a non-rebreather mask at different flow rates, 7 L/min vs. 12 L/min. We hypothesized that oxygen at a higher flow rate (12 L/min) might be more effective for the treatment of cluster headache attacks.

## Methods

### Study design

The study was designed as a double-blind, randomized, crossover study, in which patients used 100% oxygen at a flow rate of 7 L/min and 12 L/min.

The study was approved by the Medical Ethics Board of Zuyderland Medical Center Heerlen, the Netherlands. The study was registered with the European Union Clinical Trials Register (nr. 2012–003648-59) and the Dutch Trial Register (NTR3801). Written informed consent was obtained from all patients. The authors had full access to all data.

All newly diagnosed CH patients and known CH patients aged 18–65, who were naïve to oxygen treatment could be included in the study. Exclusion criteria were previous oxygen usage, pregnancy or lactation, chronic obstructive pulmonary disease, other primary or secondary headache diagnoses or other distracting painful conditions, which could interfere with the patient’s pain perception, incapacitation to understand and sign informed consent, and other contraindication for oxygen therapy as determined by the patient’s physician. If patients with secondary CH were included before imaging was conducted, they were excluded afterwards, when they were diagnosed as a secondary CH.

Patients were included with the aid of an oxygen supplier in the Netherlands. All patients were diagnosed with cluster headache by their treating neurologist, according to the ICHD-2 criteria. If the treating neurologist chose to start oxygen treatment and the patient was interested in the study, we were informed by the oxygen supplier. Patients were then contacted by us to give further details. After informed consent participants were randomized. Randomization was conducted via a random-number generator and we used a blocked-randomization design. An independent investigator kept the randomization key, which was only shared with the researchers when performing the final analysis. An oxygen tank was delivered to all patients with two covered valves, labeled valve A and valve B. Both oxygen valves were modified, so that it was not possible to change the flow rate and the flow rate itself was not visible either. One valve was set at a flow rate of 7 L/min, the other at 12 L/min. Patients as well as investigators were blinded for the flow rates. Patients were randomized into 4 groups: AB ABBA, BA ABBA, AB BAAB and BA BAAB. The first two treatment periods lasted only 1 day. Each of the other treatment periods lasted for 3 days.

As an example, the AB ABBA-scheme meant: use of valve A on day 1, B on day 2, A on days 3–5, B on days 6–8 and on days 9–11 and A on days 12–14. The treatment periods were independent of the number of attacks that occurred during each period. After completing the study, patients continued the treatment with oxygen in concordance with the dosage prescribed by their own neurologist. All patients used the same type of non-rebreather mask with a reservoir (Salter Labs E-8140).

At the start of the study patients were asked to fill in a questionnaire about characteristics of their CH attacks, medication usage (preventive medication and previously used acute treatments), and other patient characteristics. During the treatment period patients were asked to fill in a diary, in which they described, for each attack, the time until the start of oxygen treatment, pain scores before and following treatment, how long the oxygen treatment lasted, and any side effects using valve A or B. Following the 14-day study period or at the end of the cluster period, patients were asked to fill in a final questionnaire. This included questions about whether or not they had noticed any difference between flow rate A and B and if so, what that difference was and which flow rate they thought was most effective.

Patients were instructed to start treatment as soon as possible after onset of the CH attack. They had to continue oxygen treatment until the CH attack had ended or for at least 15 min. After 15 min patients were allowed to use rescue medication, usually a sumatriptan injection or nasal spray. If patients already used preventive medication (usually verapamil) at the start of the study, they were allowed to continue this medication. In order not to affect the evaluation of the primary endpoint, patients were not allowed to change the dosage or start new preventive medication until after the first 2 days of the study.

### Endpoints

The main endpoint of the study was the percentage of attacks, in which a painfree state was achieved after 15 min of treatment with oxygen, in at least 2 attacks/day on the first 2 days. Patients were asked to rate their pain before and after 15 min of oxygen treatment on a 5-point scale: 0 for pain free, 1 for mild pain, 2 for moderate pain, 3 for severe pain, 4 for very severe pain.

The secondary endpoints were, percentage of attacks treated successfully (defined as drop in VAS score of over 50%), percentage of attacks after which patients were painfree, absolute drop in VAS-score, and the patient preference to the flow rates of 7 or 12 L/min in all treatment periods. Patient preference was visualised on a 11-point scale using score − 5 for maximal preference to flow rate A and + 5 for maximal preference to flow rate B. Moreover, they were asked to choose either A, B or equal.

Attacks treated with oxygen that occurred within 12 h of other attack treatment (usually triptans) were excluded from all analysis, as these results might be confounded by the ongoing effect of that treatment. Attacks occurring within 3 h of oxygen treatment were excluded as well, as pain scores might be less reliable, when measured shortly after a previous attack.

### Sample size calculation

As studies comparing the effect of oxygen at 7 or 12 L/min have not been conducted, we did not have reliable data for conducting a sample size calculation. Therefore, we looked at the average drop in VAS score using different flow rates in our prospective study on oxygen treatment and CH [6]. Based on these results we would need to study 100 pairs of subjects or 100 subjects in a crossover design to be able to reject a null hypothesis that the response difference is zero with a probability (power) of 0.8. The type I error probability associated with the test of this null hypothesis is 0.05. It is important to note that these data do not represent our primary endpoint, but one of our secondary endpoints. Furthermore, these data were obtained in a small population and patients were not randomized between the two different flow rates. We decided to include 110 patients. This number allowed for a drop-out rate of 9.1%.

### Statistics

It was not necessary that patients completed the entire crossover design as described in the protocol, but patients could be included in the analysis if they treated attacks with both flow rates. If patients dropped out before using both treatments, or if all attacks using one flow rate had to be excluded, they were not included in the primary and secondary endpoints. As our data were not normally distributed, we had to apply non-parametric tests.

For patient preference we used a One Sample Wilcoxon Signed Rank Test and Chi square test. We calculated the percentage of successfully treated attacks, the percentage of attacks after which patients were painfree, and the average drop in VAS score, for each flow rate in every individual patient. Additionally we calculated the average drop in score on the 5-point scale. For these outcome measures we used a test for paired samples, the Wilcoxon Signed Ranks Test. To test if there was a difference between randomization groups, we used the Kruskal-Wallis Test.

We conducted an additional exploratory analysis, in which all eligible treated attacks were included. As this analysis included patients, who used only one flow rate, we used statistical tests for unpaired data. The odds ratio of treating an attack successfully or being painfree after an attack was calculated using the Chi square test and the Fisher’s exact test. We used a independent samples T-test for the drop in VAS-score and drop in score on the 5 point-scale.

All statistical tests were 2-tailed and the significance threshold was set at 0.05. All statistical analyses were performed using SPSS.

## Results

In total we included 98 patients, from 28 Dutch centers, between March 2013 and October 2016. The initial goal was to include 110 patients. Patient recruitement, however, was slower than expected and we had to stop inclusion before reaching our goal.

**Fig. 1:**
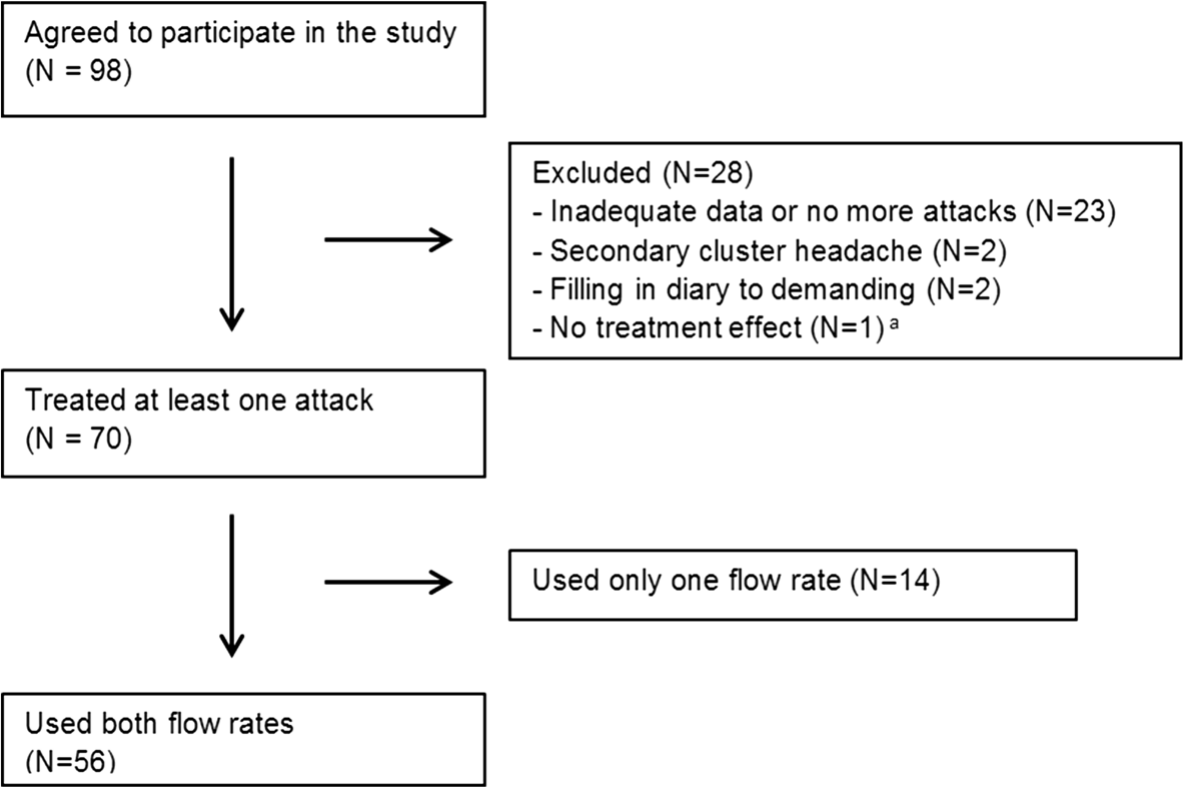
Flowchart of the study population ^a^One patient did not notice any effect on start of the treatment and did not want to participate (we did not receive data of these first attacks)

Out of 98 patients, 28 had to be excluded (Fig. 1). We did not receive adequate data (questionnaires and diary) of most of these patients. We were not always able to identify the reasons for not participating in the study, but the most common reason was that cluster headache attacks did not occur anymore (end of the cluster period). There were 2 cases of secondary CH (1 intracerebral arteriovenous malformation located just cranial to the tectum and 1 pituary tumour). Of the 70 patients who provided adequate data, 56 patients used both treatments. Table 1 shows the baseline characteristics of these 56 patients. These patients treated a total of 680 attacks, of which 76 attacks had to be excluded, leaving 604 attacks for the analysis.

**Table 1:** Baseline data. *N* = 56

Gender	61.1% male - 38.9% female
Age (median – range)	43.0 (20–76)
Current or past smoker	74.1% (50% current - 24.1% past)
Painfree periods (episodic) (*N* = 31)	67.7%
Pain between attacks	50.9%
Attack duration untreated (median-range)	60 min (9–330)
Attack frequency (median-range)	2 attacks per day (0.1–8)
Verapamil usage	55.4%

Because most patients were recently diagnosed with CH, and often were experiencing their first cluster period, it was not always possible to differentiate between chronic or episodic CH. Of 31 patients who answered this question, 67.7% reported having episodic CH. Five patients had an average attack duration of longer than 180 min and 3 patients had less than one attack in 2 days. As all of the patients, who did not fulfil the duration or frequency criterium, had individual attacks that met the ICHD-2 criteria [7] and otherwise had typical symptoms apart from frequency or duration, the most likely diagnosis remained CH. As reported in a previous study, these patients were not excluded from the analysis [8]. All patients had at least one autonomic symptom or reported restlessness during attacks.

### Side effects

No serious side effects were reported in either treatment group and there was no significant difference in the occurrence of these side effects between treatment groups (Table 2). All patients were able to continue treatment despite of these side effects.

**Table 2:** Side effects

Side effects	7 L / min (*n*)	12 L /min (*n*)
Lightheadedness	10	11
Dry mouth	5	6
Tired after treatment	2	2
Difficulty breathing^a^	2	1
Nasal congestion	1	1
Coughing	1	1
Difficulty sleeping after treatment	0	2
Nausea	1	1
Hoarse voice	0	1
Tingling	1	0
Burning eyes	1	0
Cold feeling	1	0
Blurry vision	0	1

^a^Severity not specified, both patients were able to continue treatment

### Primary outcome measure

Only 5 patients met the inclusion criteria for our primary outcome measure; two treated attacks on each of the first 2 days. These patients treated a total of 27 attacks on the first 2 days. No significant difference was found in the percentage of attacks after which patients were painfree between 7 and 12 L/min (*p* = 0.180) (Table 3).

Looking at the individual attacks, the odds ratio (Fisher’s exact test) for being painfree after 15 min of treatment was 3.75 (95% CI 0.58–24.28), favouring 12 L/min (*p* = 0.209). However, as numbers were so small, there was clearly inadequate power.

**Table 3:** Results primary and secondary endpoints

	Included (N)	Results (Median*+*IQR^a^)	*P*-value
% Painfree after treatment (first 2 days)	5	7 L/min: 0 (0–37.50)	0.180^b^
		12 L/min: 0 (0–83.50)	
Drop in VAS score	56	7 L/min: 4.09 (2.67–5.48)	0.243^b^
		12 L/min: 4.33 (2.71–5.41)	
% Successfully treated attacks	56	7 L/min: 92.86 (45.10–100)	0.505^b^
		12 L/min: 84.52 (33.33–100)	
Patients preference (0–10)	46	3.50 (0.50–5.50)	0.005^c^
0 favouring 12 L/min			
10 favouring 7 L/min			
Patients preference	49	7 L/min: *N* = 11	0.059^d^
7 L/min - 12 L/min - equal		12 L/min: *N* = 24	
		Equal: *N* = 14	
% Painfree after treatment (all attack periods)	55	7 L/min: 28.57 (0–66.67)	0.039^b^
		12 L/min: 0 (0–50.00)	
Drop on 5-point scale	55	7 L/min: 1.50 (1–2)	0.475^b^
		12 L/min 1.50 (1–2)	

^a^Median + interquartile range (25–75)

^b^Wilcoxon Signed Ranks Test

^c^One sample Wilcoxon Signed Rank Test

^d^Chi square test

### Secondary outcome measures

The percentage of attacks treated successfully and absolute drop in VAS-score showed no significant difference between both treatment groups (Table 3). Patient’s preference at the end of the study, expressed on a scale of 0–10, showed a median score of 3.5 (favouring 12 L/min). This was statistically significant (*p* = 0.005). Twenty-four out of 49 patients chose 12 L/min over 7 L/min, compared to 11 patients who preferred 7 L/min (*p* = 0.059). Contradicting this result, a higher percentage of attacks after which patients were painfree, as scored on a 5-point scale, was found in the 7 L/min group. This was statistically significant (*p* = 0.039). The average drop in score on this 5-point scale was equal between groups.

### Missing data

Ten patients used both treatments but did not answer the question considering preferred valve. There were no significant differences in these 10 patients on other outcome measures.

Fourteen patients only used one treatment; 6 used 12 L/min and 8 used 7 L/min. We conducted an exploratory analysis, in which all eligible attacks in 70 patients were included. Seven hundred ten attacks were reported, of which 78 had to be excluded, leaving 632 attacks for the analysis. Table 4 gives an overview of all attacks included in this analysis. No significant differences were found between groups. However, the likelihood of being painfree was lower using 12 L/min, OR 0.73 (95% CI 0.52–1.02), which was nearly statistically significant (*p* = 0.061). The average drop on the 5-point scale was equal between groups.

**Table 4:** Overview of all attacks treated in 70 patients

	7 L/min	12 L/min	Odds ratio (95% confidence interval)	*P*-value
Patients	64	62		
Total number of attacks treated	344	366		
Attacks excluded^a^	37 (10.8%)	41 (11.2%)		
Attacks included in analysis	307	325		
Attacks / patient (range)	4.80 (1–19)	5.24 (1–17)		
Successfully treated	211 (68.7%)	219 (67.4%)	0.94 (0.67–1.31)	0.717^*c*^
Drop in VAS score (Mean – SD)	4.23 SD 2.06	4.28 SD 1.75		0.734^*d*^
Painfree after treatment^*b*^	109 (36.2%)	93 (29.2%)	0.73 (0.52–1.02)	0.061^*c*^
Drop on 5-point scale (Mean – SD)^*b*^	1.61 SD 0.77	1.63 SD 0.66		0.683^*d*^

^a^Attacks were excluded because of occurrence within 3 h of previous oxygen treatment or within 12 h of other attack treatment

^b^No data available of 6 attacks treated with both 7 L/min and 12 L/min

^c^Chi square test

^d^Independent samples T-test

### Randomization effect

No significant differences were found between randomization groups on success or painfree percentages and patient preference. Considering the drop in VAS score, randomization group 4 showed a larger drop in VAS score using 12 L/min compared to group 3, which showed a larger drop in VAS score of 7 L/min. This showed statistical significance. On all other outcome measures, group 3 and 4 were equal to the other groups.

## Discussion

Our primary outcome did not show a significant difference between 7 L/min and 12 L/min. As this was based on a small number of patients, we feel that this result is of little clinical significance. The results of our secondary outcome measures are somewhat conflicting. More patients seem to be painfree using 7 L/min, although the absolute differences are small (29.2 vs 36.2%). We could not confirm this trend in our other outcome measures and contradicting these results, patients tended to favour 12 L/min.

**Fig. 2:**
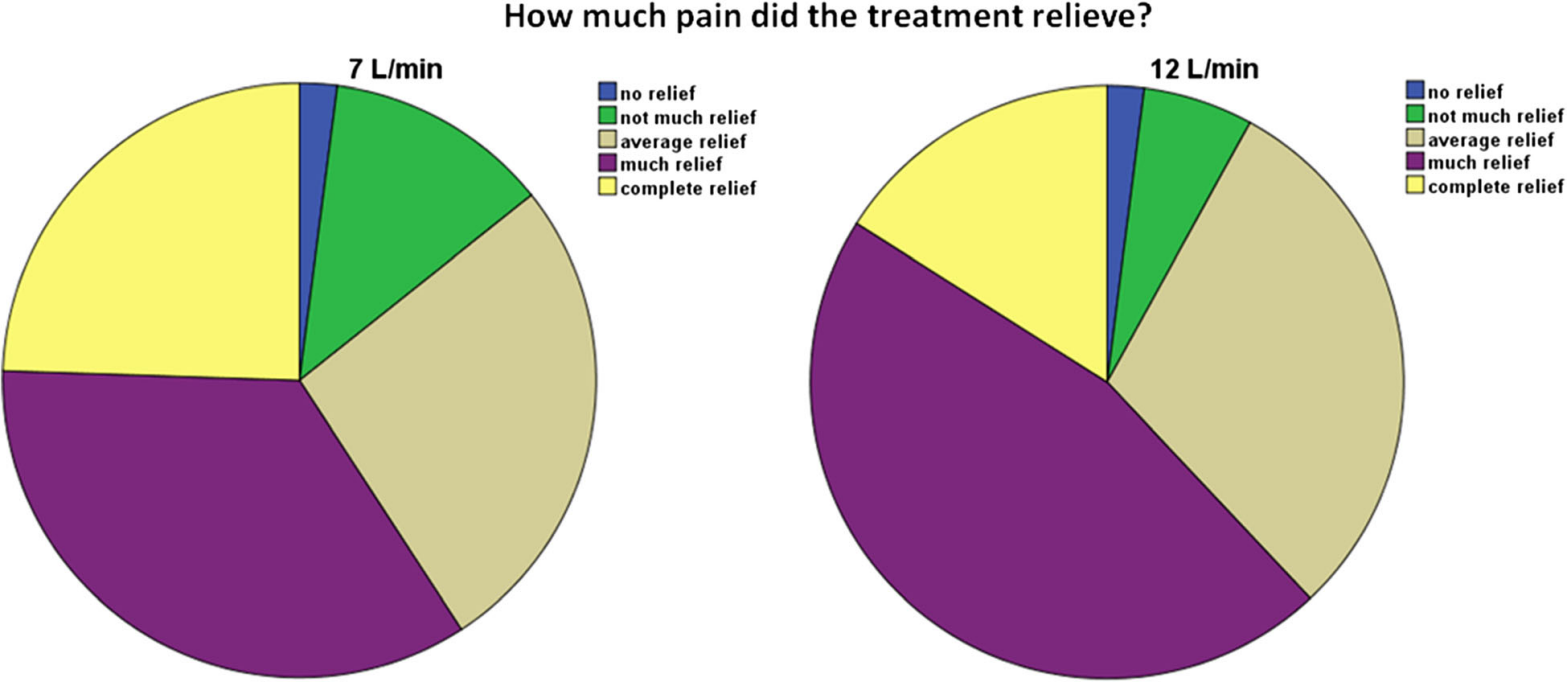
How much pain was relieved on average? *N* = 49

**Fig. 3:**
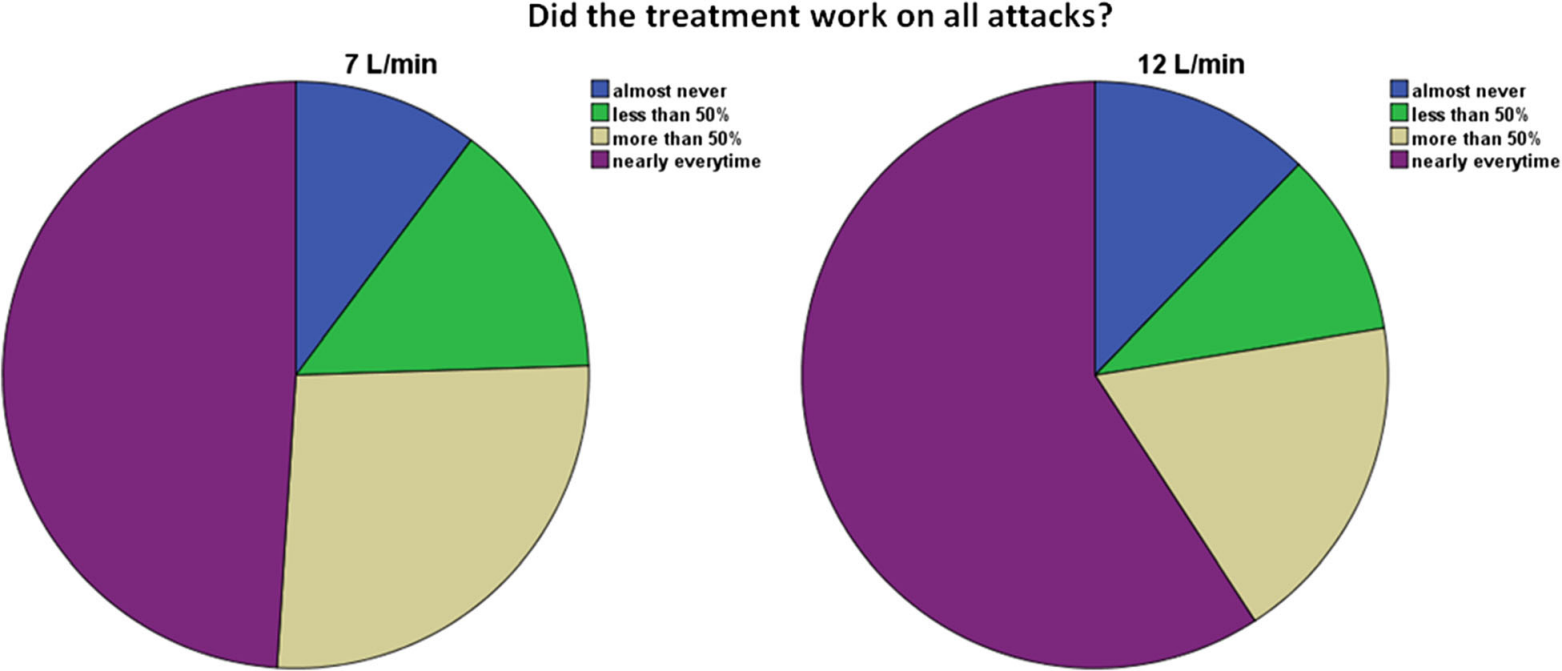
Was the treatment effective in all attacks? *N* = 49

The average drop in score on the 5- point scale was equal between groups. This suggests that the average response, besides the patients being painfree, was worse in the 7 L/min group. This explanation was supported by some of the questions on our final questionnaire. Figure 2 shows that slightly more patients had no or not much relief on 7 L/min. Figure 3 shows that slightly more patients found 12 L/min to be effective in all attacks. This might explain the preference for 12 L/min. These results, combined with our other outcome measures, suggest that although more patients were painfree using 7 L/min, this is insufficient to state that 7 L/min is the more effective treatment.

Patient preference scored by patients from 0 (favouring 12 L/min) to 10 (favouring 7 L/min), shows a considerable number scoring lower than 1.0 (13/46), and only 1 patient scoring higher than 9.0. Besides this difference the results are almost normally distributed. This could be interpreted in a way that there seems to be a subgroup of patients, who absolutely favours 12 L/min, while in the rest of the population there does not seem to be a difference between both treatment groups. We wondered if we could identify this population of patients having an absolute preference for 12 L/min. As a considerable number of patients experienced their first attack period, we could not always differentiate between episodic or chronic CH. Five out of 9 patients in the 12 L/min preference group, compared to only 4 out of 20 in the no-preference group had chronic CH. The Chi square test did not show statistical significance, possibly due to low power, but this might be an interesting trend. No significant differences were found between episodic or chronic CH on our other secondary outcome measures. The use of verapamil was not related with a preference for one of the two flow rates. We also screened for gender, age, smoking, attack characteristics and specific autonomic symptoms or restlessness, but no significanct between group differences were found.

The most common side effect of oxygen usage was lightheadedness. This was equally found in both flow rates. Patients were instructed to breathe normally while using the oxygen. However, some patients might have been hyperventilating, which could have caused this lightheadedness.

No other controlled trials compared different oxygen flow rates for CH attacks. In previous studies oxygen at both 7 L/min and 12 L/min was shown to be effective [1, 3]. As these studies used different outcome measures they are difficult to compare. As our study did not show any difference in the occurrence of side effects between groups, we can state that there is no consistent difference in treatment effect and safety between oxygen at a flow rate of 7 and 12 L/min. An interpretation of our results may be to treat all patients with 12 L/min, as the only disadvantage would be the higher production and delivery costs. An alternative would be to treat people with 7 L/min, and if this is not successful, or not sufficiently satisfying, use 12 L/min instead, as is often current practice today.

### Methodological considerations

A crossover design was used in previous studies on oxygen treatment and CH [1–3, 9]. We chose this type of design as new or oxygen naïve CH patients are not easily recruited. An important benefit of a crossover design is that each patient receives both treatments. This eliminates confounders caused by different treatment groups and results in a smaller sample size necessary to detect a significant treatment effect. An important disadvantage of crossover designs is the potential carry over effect. As oxygen is assumed to have a very short washout period, a crossover design is suitable for a trial with oxygen treatment.

Randomization groups were not equally distributed due to drop-out and we found a significant difference in drop in VAS score between groups 3 and 4. We do not have a good explanation, why results in these groups were different, however, as group 4 was the smallest (*n* = 11) and there was a wide variation between results, this might well be a sampling error. The way the randomization scheme was built, makes it less likely that there is a significant carry over effect. More specifically, group 3 and 4 only differed on the first 2 days.

Our aim was to include 110 patients, but we were only able to include 98. Patient recruitement was slower than expected, and due to the long duration of the study, we had to stop recruitement before including 110 patients. We chose to allow for an estimated drop-out rate of 9.1%. In retrospect, this should have been considerably higher. There was a large group of patients, who agreed to participate, but eventually did not. As most patients were recently diagnosed with CH and it often takes some time before the diagnosis is made, several patients would be near the end of their cluster period at the time of inclusion. As in most of these cases, we did not receive the first questionnaire with baseline characteristics, we are not sure if this may have resulted in a bias, but there is no reason to assume this.

Fourteen patients only used one flow rate. As these were nearly equally distributed between flow rates and treatment results were not significantly different in these patients, we have no reason to assume selective drop-out. Furthermore, the exploratory analysis, including all eligible treated attacks, showed similar results.

The reason for our primary outcome was that we would find a difference before verapamil was started or the dosage increased. It did not seem ethical to exclude patients from prophylactic treatment during the 14 days of the trial. We could only include 5 patients for analysis of our primary outcome measure. There were more patients who had over 2 attacks a day on average. However, as patients mostly started oxygen treatment immediately after they received there tanks at home, day 1 of the study often started halfway through the day. As attacks often occured at night this resulted in relatively few attacks being registered on the first day. Furthermore we had to exclude attacks in some patients. In retrospect the primary outcome measure should have been chosen differently. However, we do think that our secondary outcome measures are adequately chosen. These outcome measures reflect the results of a large number of attacks and we did not find a confounding effect of verapamil usage on our results.

In our study, patients were not treated clinically and we had to rely on data delivered in a diary and questionnaires. Depending on the way patients interpreted questions and scores this may have created a bias in results. However, it is unlikely that this would create a specific bias favouring one of the two treatment methods. Patients had to note the date and timing of each attack and what valve they used. We contacted patients before the start of the study and one week after the start of the study to make sure all data was correctly noted. If there were inconsistencies, these attacks were excluded. We checked whether oxygen was used in each case (by checking the oxygen tanks), but we were unable to check if both valves had been used. This made it difficult to detect a possible violation of protocol. The blind was not broken for any patient during the study.

### Future studies

Ideally another study with more power should be conducted to see if oxygen at a flow rate of 12 L/min is consistently preferred by patients and if subgroups can be found. It is, however, difficult to recruit oxygen naïve cluster headache patients. It remains unclear which outcome measure is the most appropriate for a trial on the treatment of cluster headache.

## Conclusion

Patients preferred the treatment with oxygen at a flow rate of 12 L/min compared to 7 L/min. We did not find a significant difference in drop in VAS score or successfully treated attacks, but more patients were painfree after using 7 L/min. The preference for 12 L/min might be explained by the fact that there were more patients in which treatment with 12 L/min was effective in all attacks, and less patients in which treatment was ineffective. These results suggest, that although more patients were painfree using 7 L/min, this is insufficient to state that 7 L/min is the more effective treatment. We suggest there is a subgroup of patients, who benefit from using the higher flow rate. In this study we were unable to further define this subgroup, possibly due to small sample sizes. As no difference in side effects were found, the usage of oxygen at a flow rate of 12 L/min is at least equally safe as 7 L/min and could be used in all patients. From an economic perspective it might be more cost-effective to start treatment with 7 L/min and if ineffective to switch to 12 L/min. This, however, remains open for debate.

## Abbreviations

CH: Cluster headache
CI: Confidence interval
IQR: Interquartile range
OR: Odds ratio
